# Exploring gene networks in two sunflower lines with contrasting leaf senescence phenotype using a system biology approach

**DOI:** 10.1186/s12870-019-2021-6

**Published:** 2019-10-24

**Authors:** Sebastián Moschen, Johanna Marino, Salvador Nicosia, Janet Higgins, Saleh Alseekh, Francisco Astigueta, Sofia Bengoa Luoni, Máximo Rivarola, Alisdair R. Fernie, Nicolas Blanchet, Nicolas B. Langlade, Norma Paniego, Paula Fernández, Ruth A. Heinz

**Affiliations:** 1 Estación Experimental Agropecuaria Famaillá, Instituto Nacional de Tecnología Agropecuaria, Famaillá, Tucumán Argentina; 2 Instituto de Agrobiotecnología y Biología Molecular – IABiMo – INTA-CONICET, Instituto de Biotecnología, Centro de Investigaciones en Ciencias Veterinarias y Agronómicas, Instituto Nacional de Tecnología Agropecuaria, Hurlingham, Argentina; 3 Consejo Nacional de Investigaciones Científicas y Técnicas, Ciudad Autónoma de Buenos Aires, Argentina; 4 Escuela de Ciencia y Tecnología, Universidad Nacional de San Martín, San Martín, Argentina; 5 Earlham Institute, Norwich Research Park, Norwich, NR4 7UZ UK; 6 Max-Planck-Institut für Molekulare Pflanzenphysiologie, Potsdam-Golm, Germany; 7 Instituto Tecnológico Chascomús (INTECh), Consejo Nacional de Investigaciones Científicas y Técnicas (CONICET)-Universidad Nacional de General San Martín (UNSAM), Chascomús, Argentina; 8 LIPM, INRA, CNRS, Université de Toulouse, Castanet-Tolosan, France

**Keywords:** Sunflower, Leaf senescence, Candidate genes, Functional genomics, System biology

## Abstract

**Background:**

Leaf senescence is a complex process, controlled by multiple genetic and environmental variables. In sunflower, leaf senescence is triggered abruptly following anthesis thereby limiting the capacity of plants to keep their green leaf area during grain filling, which subsequently has a strong impact on crop yield. Recently, we performed a selection of contrasting sunflower inbred lines for the progress of leaf senescence through a physiological, cytological and molecular approach. Here we present a large scale transcriptomic analysis using RNA-seq and its integration with metabolic profiles for two contrasting sunflower inbred lines, R453 and B481–6 (early and delayed senescence respectively), with the aim of identifying metabolic pathways associated to leaf senescence.

**Results:**

Gene expression profiles revealed a higher number of differentially expressed genes, as well as, higher expression levels in R453, providing evidence for early activation of the senescence program in this line. Metabolic pathways associated with sugars and nutrient recycling were differentially regulated between the lines. Additionally, we identified transcription factors acting as hubs in the co-expression networks; some previously reported as senescence-associated genes in model species but many are novel candidate genes.

**Conclusions:**

Understanding the onset and the progress of the senescence process in crops and the identification of these new candidate genes will likely prove highly useful for different management strategies to mitigate the impact of senescence on crop yield. Functional characterization of candidate genes will help to develop molecular tools for biotechnological applications in breeding crop yield.

## Background

Senescence is the final stage of leaf development preceding cell death and is also a complex process controlled by multiple genetic and environmental variables. Once the senescence program is triggered, highly regulated gene expression changes occur, leading to profound changes in leaf metabolism [1–4].

Senescence is an active phase of plant development involving regulated degradation and remobilization processes which could reduce crop yield when it is induced prematurely [5]. Annual plants, which include many of our grain and oil crops, undergo a visual process towards the end of the reproductive stage that is accompanied by nutrient remobilization from leaf to developing seeds [1].

Sunflower is one of the most important oil crop worldwide. A delay in leaf senescence has been directly correlated to grain yield, mainly due to photosynthesis maintenance during the reproductive stage [6–9].

The study of leaf senescence has been greatly advanced in the past two decades by the availability of a wide range of complementary *omics* technologies allowing the study of this complex phenomenon by a holistic phenomics approach [10]. Recently, Kim et al. [11] performed a revision of milestones in leaf senescence research obtained using multi-omics technologies, highlighting the importance of this analysis to address unresolved questions regarding to this process.

Recent efforts mean that functional genomics tools for cultivated sunflower involving physiological, transcriptional and metabolic profiles are now available [12–20].

That said, the major current focus of senescence research has been conducted on leaves in the model plant species Arabidopsis. However, knowledge underlying functional and regulatory mechanisms of senescence in other organs and/or in other species, especially agriculturally relevant crop, is still limited [10].

Gene expression profiling has been carried out during natural and induced senescence, taking advantage of recent progresses in Next-Generation Sequencing (NGS) and transcriptome analysis [21–23].

Recently, using an integrative metabolomic and transcriptomic approach, Li et al. [24], studied the remobilization of nutrients from senescing leaves to sink tissues in tobacco. In this study, metabolic pathways associated to tricarboxylic acid cycle and related metabolism of sugars, amino acids, and fatty acids were upregulated. This results highlight the energy metabolism as a relevant pathways during leaf senescence process.

In our previous study, we applied microarray technology [16] to study leaf senescence progress in a commercial sunflower hybrid, analyzing the transcriptomic and metabolic profile in a system biology approach [19, 25]. This results allowed the identification of several candidate genes involved in the leaf senescence program in this crop.

Considering the importance and the need to characterize inbred lines for sunflower breeding, we have recently performed a selection of contrasting sunflower lines for leaf senescence progress under field conditions through a physiological, cytological and molecular approach [20]. In this study, we identified and characterized the two most contrasting inbred lines from the INTA Association Mapping Population with respect to leaf senescence [26], R453 and B481–6, displaying early and delayed senescence phenotypes, respectively. These advancements, concomitant to the recent release of the sunflower genome [27], enabled us to take the present work to the systems biology level by integrating large scale transcriptomic analysis with metabolic profiling of the contrasting sunflower inbred lines. In the current study we identify both metabolic pathways associated with leaf senescence in sunflower as well as putative transcription factors that represent useful candidate genes for crop breeding.

## Results

**Fig. 1:**
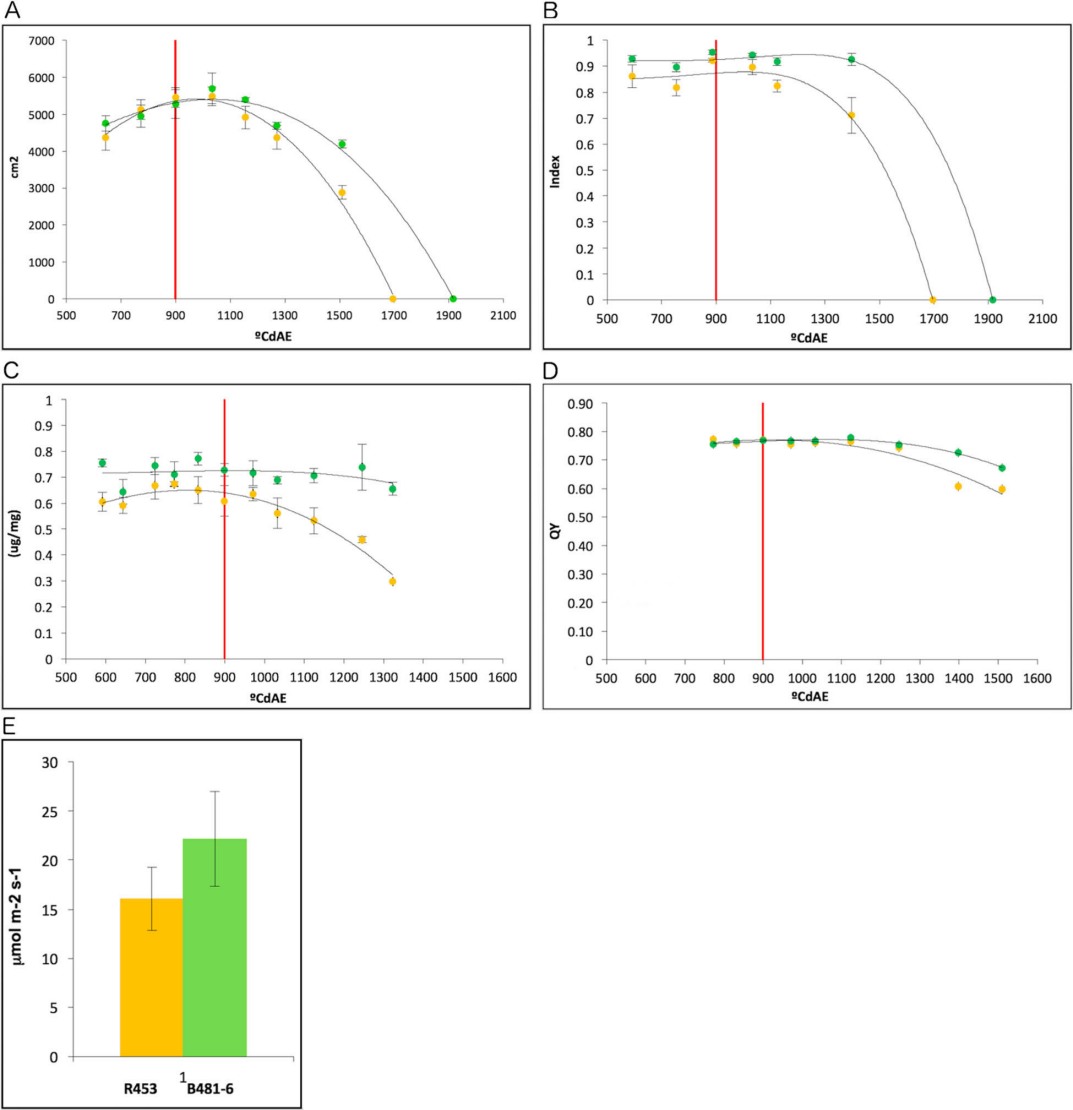
Physiological measurements of senescence progress during field experiment at INTA Castelar, Argentina. **a** Leaf area; **b** Interception radiation index in the canopy; **c** Chlorophyll content of leaf 15; **d** Quantum yield of photosystem II of leaf 15; **e** Photosyntesis rate of leaf 15. Orange and green dots represent R453 and B481–6 line respectively. Tendency lines are in polynomic function. The red line indicates anthesis time. Error bars correspond to standard errors. °CdAE indicate thermal time (°C days After Emergence)

Physiological measurements were performed to evaluate differences in the progress of senescence process between the lines. Green leaf area (GLA) showed similar progress until 100 °Cd after anthesis (1000 °CdAE). At this point, the GLA in the early senescence line, R453, decreased abruptly and reached zero at 1700 °CdAE, while the delayed senescence line, B481–6, displayed a gradual progression of senescence only reaching absolute senescence at 1900 °CdAE (Fig. 1a). Moreover, at the canopy level, B481–6 line showed a maximum interception radiation index until 1500 °CdAE whereas, in R453, the intercepted radiation began to decrease rapidly after anthesis (Fig. 1b).

Both lines presented similar architecture, same anthesis time (899 °CdAE) and an average of 25 leaves per plant. To perform molecular analysis, we selected leaf 15 numbered from the bottom to the top of the plant. Chlorophyll content in this leaf was similar until anthesis time. At this moment, the chlorophyll content of R453 started to decrease whereas that in B481–6 remained stable at about 0.7 μg/mg (Fig. 1c). Quantum yield efficiency of photosystem II presented an analogous evolution during leaf development, showing a higher decrease after anthesis in the early senescence line (Fig. 1d). Twelve days after anthesis, the photosynthetic activity was measured in leaf 15 (Fig. 1e). Despite not having a significant difference, B481–6 showed higher photosynthesis activity tendency respect to R453 which is coherent with a delay in senescence program.

**Fig. 2:**
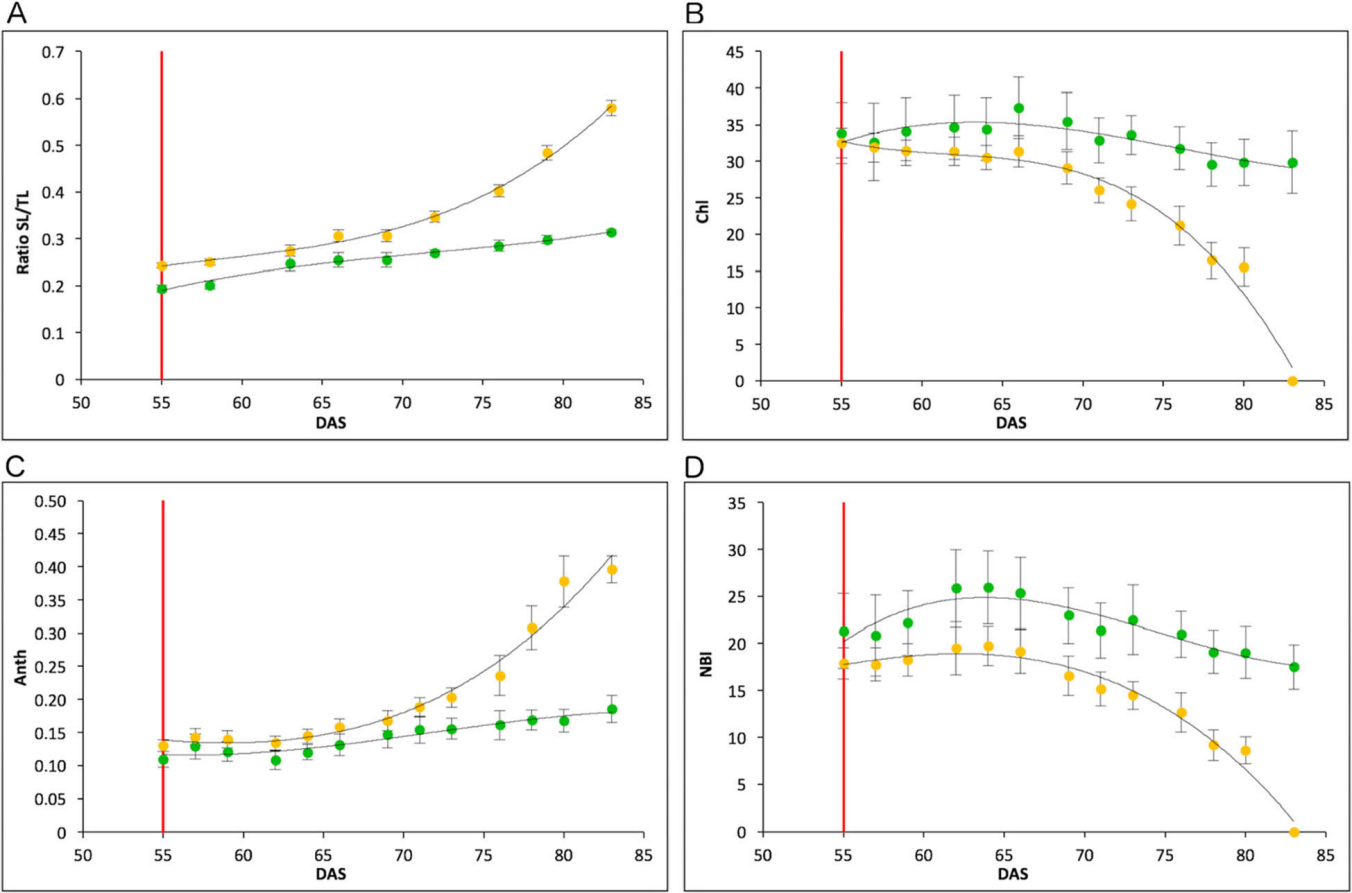
Physiological measurements of senescence progress during field experiment at Heliaphen Platform, INRA Toulouse, France. **a** Ratio of senescence leaf number vs. total leaf number; **b** - **d** Dualex measurement of chlorophyll content index (**b**), anthocyanin content index (**c**) and Nitrogen Balance Index (**d**). Orange and green dots represent R453 and B481–6 line respectively. Tendency lines are in polynomial function. The red line indicates anthesis time. Error bars correspond to standard errors. DAS: Days After Sowing

With the aim of characterizing both lines in a different environment we performed a phenotyping experiment on the Heliaphene platform at INRA – Toulouse, France [28]. In this experiment, differences in both lines after anthesis were similar to the previous results. The ratio Senescent Leaf / Total Leaf showed an early increase in R453 while B481–6 remained constant and displayer a slower rate of senescence (Fig. 2a). We also measured chlorophyll, anthocyanin and Nitrogen balance index (NBI) on leaf 15 (Dualex Scientific+). Regarding the chlorophyll index, R453 line presented a strong decrease 10 days after anthesis and reached zero 28 days after anthesis. At this time point, leaf 15 in B481–6 maintained higher levels of chlorophyll, close to the maximum (Fig. 2b). The anthocyanin index increased during leaf senescence in both lines showing a similar rate until 15 days after anthesis. At this time point, anthocyanin content rapidly increased in R453 but only slowly in B481–6 (Fig. 2c). The nitrogen balance index showed a maximum 10 days after anthesis, at this point, NBI started to decrease at a higher rate in R453 than B481–6 which remained stable even until 30 days after anthesis (Fig. 2d).

### RNA-seq analysis

**Fig. 3:**
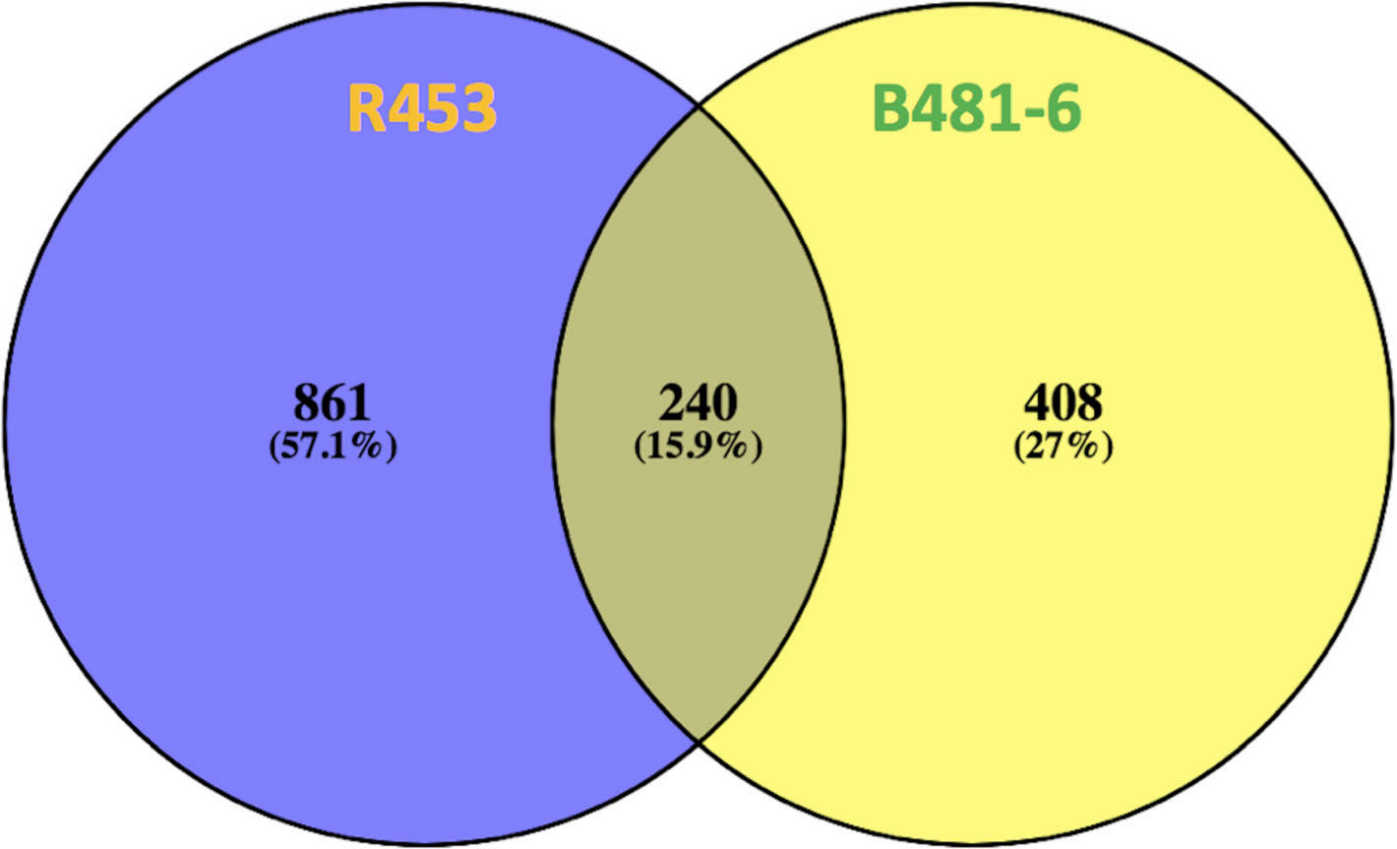
Venn diagram of differential expressed genes. 1101 and 648 genes were deferentially expressed in R453 and B481–6 respectively at Post-anthesis vs. Anthesis time

With the aim to evaluate differences in the leaf senescence program induced by anthesis, differential expression analysis was performed. This analysis showed 1101 and 648 genes differentially expressed between post-anthesis vs. anthesis in the early and delayed senescence lines respectively. Additionally, we compared these lists of genes, and found 240 genes shared by both lines. In addition, a total of 861 genes were differentially expressed only in R453 and 408 genes were differentially expressed only in B481–6 (Fig. 3). The 240 genes shared by both lines displayed the same expression pattern, although most of them presented higher fold-changes in the early senescence line (R453). Regarding the genes differentially expressed only in R453, we found higher expression levels of genes related to transporter families, cell wall and lipid degradation, protein degradation and redox homeostasis but lower expression levels of genes related to cell cycle, RNA transcription, secondary metabolism and genes related to auxin metabolism and macromolecules synthesis. Meanwhile, the list of genes differentially expressed only in B481–6 displayed higher expression levels of genes related to heat shock proteins, light signalling, signalling receptor kinases, biotic and abiotic stress response and lower expression levels of genes related to transport and some genes related to lipid metabolism (Additional file 3: Table S1).

### Primary metabolite analysis

Using GC-MS, we detected 71 metabolites including different amino acids, organic acids, sugars and sugar alcohols (Additional file 4: Table S2). Carbohydrate and tricarboxylic acid cycle intermediate levels decreased during post-anthesis senescence in both lines (Additional file 1: Figure S1). Lysine, proline, aromatic (tryptophan, tyrosine, and phenylalanine) and branched-chain (isoleucine and valine) amino acids and some amino acid derivatives from secondary metabolism increased during senescence. Asparagine - a metabolite associated with nutrient recycling - levels increased during leaf senescence in the early senescence line (Additional file 1: Figure S1a).

### Integration analysis

**Fig. 4:**
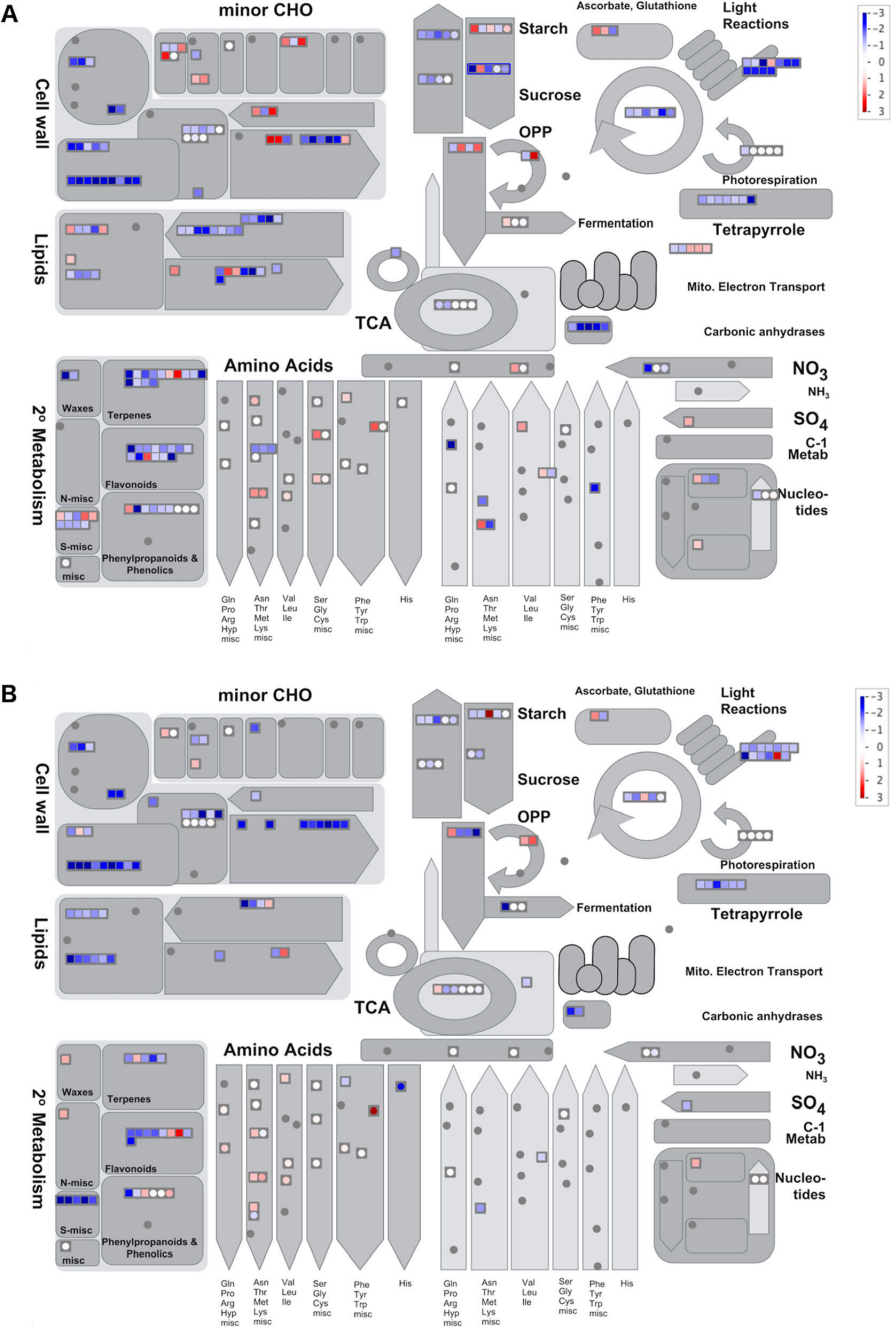
Metabolism overview using Mapman. Post-anthesis vs. Anthesis in **a** early senescence line (R453), and **b** delayed senescence line (B481–6). Genes and metabolites are represented by squares and circles, respectively. Colour intensity corresponds to the expression ratio at logarithmic scale (log2). Red: upregulated, blue: downregulated

Mapman analysis [29] was performed to integrate transcriptomic and metabolic data and to compare the evolution of post-anthesis senescence in both lines. When comparing the most altered biological processes during leaf senescence, we found downregulation of genes and metabolites associated to photosynthesis and photorespiration, cell wall modification, starch and sucrose synthesis in both lines (Fig. 4). Whereas most of genes related to catabolic process remained at lower expression levels in the delayed senescence line, the early senescence line showed upregulation of genes associated with sugar and starch degradation. Meanwhile, genes related to cell wall and lipid degradation and genes associated with the synthesis of amino acids related to nutrient recycling showed an early activation of the senescence program in the early leaf senescence line.

### Transcription factor analysis

**Fig. 5:**
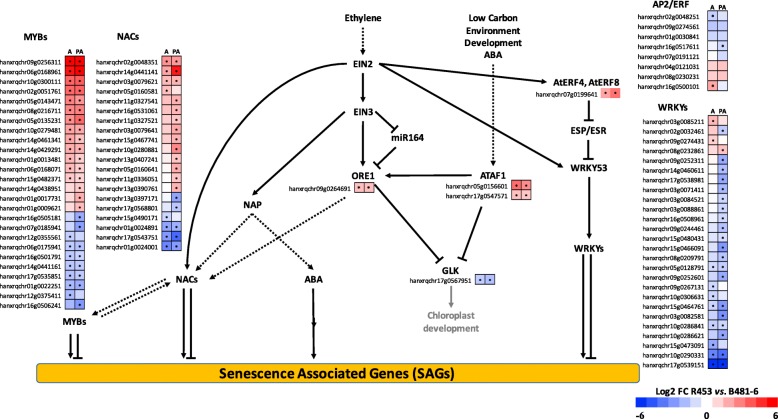
Transcription factor analysis of ethylene signalling pathway and the associated TFs families. The squares indicate the expression ratio at logarithmic scale (log2) of early (R453) vs. delayed senescence line (B481–6) at anthesis (A) and post-anthesis (PA). Asterisk indicate significant differences, p-*adj* < 0.05

Transcription factors (TFs) are key regulators of the different biological processes identified with the metabolomics and global transcriptomic approach. Ethylene signalling pathway and TFs belonging to NAC, MYB and WRKY families have been widely reported as being senescence-associated in many different plant species and constitute obvious candidate genes to focus on and identify the leaf senescence regulatory network [18, 30–33]. In this work, we compared the expression levels of all the TFs from these families annotated in the sunflower genome comparing R453 vs. B481–6 at anthesis and post-anthesis to identify differences between the lines. Members of the ethylene signalling pathway *ORE1* and *ATAF1* were upregulated in R453 in comparison to B481–6 at both time points, whereas *GLK,* a negatively regulated gene by those NACs members, was downregulated (Fig. 5). The pathway downstream of these genes involves many different NAC, MYB and WRKY TFs. In this analysis, we found that most of the NAC and MYB TFs detected with significant differences in the differential expression analysis were upregulated (and mostly to a large extent) in the early senescence line. By contrast, most of the WRKY TFs were downregulated in this line (Fig. 5).

**Fig. 6:**
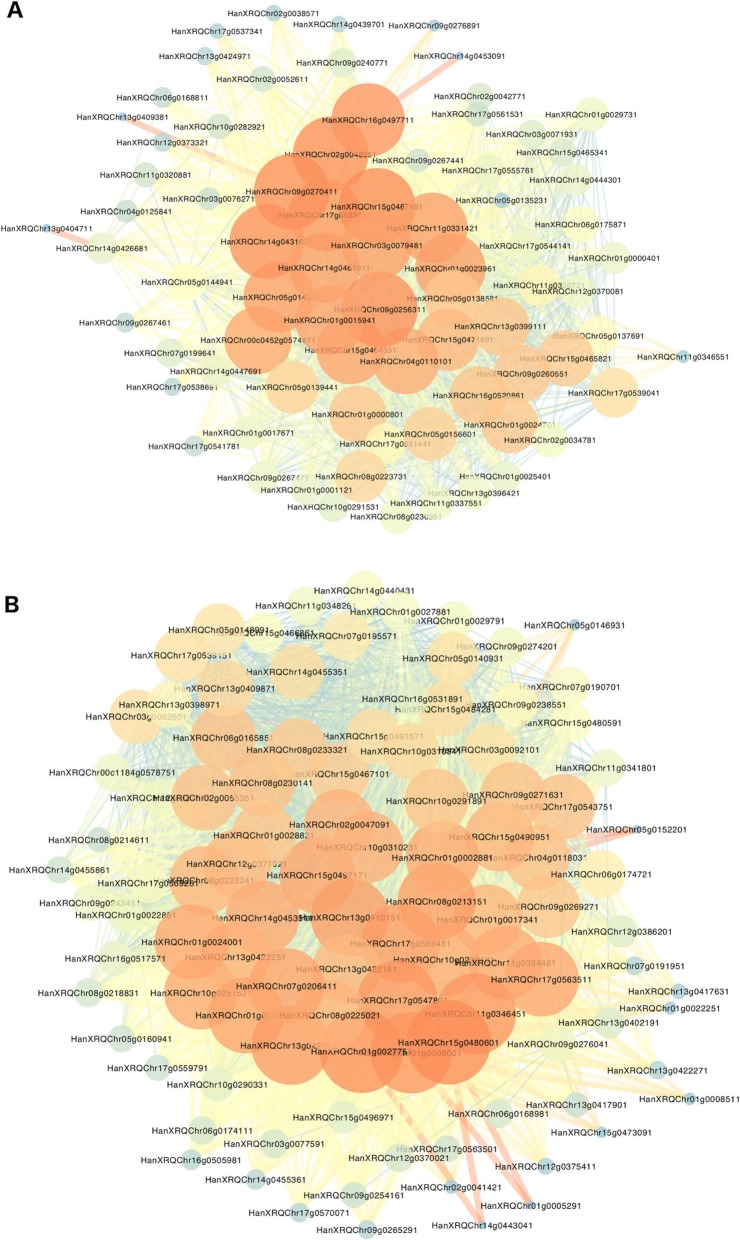
Gene co-expression networks analysis (WGCNA). **a** upregulated TFs and **b** downregulated TFs at anthesis time, R453 vs. B481–6 with Log2 fold-change > 2 or < − 2. The nodes represent TFs and the edges represent connections between them. The node size and colour are related to the number of connections, large orange nodes represent highly connected hub genes (high degree), small blue nodes represent genes with few connections (low degree). Strong connections are visualized as wider lines

With the aim to find hub genes that act as main regulators of early development related leaf senescence, we performed a WGCNA analysis focusing on the TFs differentially expressed at anthesis time, R453 vs. B481–6 (Log2 fold-change > 2 or < − 2). Two different networks were generated for easy visualization, namely upregulated hubs and downregulated hubs (Fig. 6a and b). The *degree* parameter - defined as the sum of connection strengths with the other network genes - was used to identify *hubs* genes. The upregulated network displayed 79 nodes and 1373 edges; 25 genes were reported as hubs with a degree higher than 50. Most of them showed higher expression levels in the early senescence line and corresponded to TFs from NAC MYB and WD40-like families (Additional file 5: Table S3). The downregulated network contained 102 nodes and 3014 edges; 36 genes were reported as hubs with a degree higher than 80. Most of those genes displayed lower expression levels in the early senescence line at anthesis (but were upregulated in the delayed senescence line), and the most represented families were the bHLH, C2H2, NAC and CCHC (Zn) families (Additional file 5: Table S3).

## Discussion

In different crops, a delay in leaf senescence may have an important impact on grain yield through the maintenance of the photosynthetic leaf area during the reproductive stage [5]. In sunflower, this process is critical due to the incapacity of the current hybrids to keep their green leaf area after anthesis [6, 7]. The study of the senescence process in crops is complex because of several levels of temporal and spatial dynamics and the different biotic and abiotic stresses [10]. Therefore the integration of multi-omics approaches in association with physiological phenotyping appears to be necessary to enable the dissection of the complex phenomenon of senescence [10, 11].

In several different crops, senescence is induced in the whole plant after flowering and is controlled by the development of reproductive structures [5, 34]. In this study, we characterized two sunflower inbred lines contrasting in their leaf senescence after anthesis and performed a transcriptomic and metabolomic analysis to understand the metabolic pathways underlying this process with the aim to identify genetic pathways amenable for crop improvement.

Both lines, R453, and B481–6, presented similar architecture and phenology until anthesis, the same anthesis time and an average of 25 leaves per plant. The main differences in these lines are related to the progress of leaf senescence after anthesis as previously reported by our group [20]. In this work, we performed a phenotyping assay in two different environments to evaluate the robustness of these differences. Both experiments presented similar results with early activation of senescence process after anthesis in R453, showing in this line an early increase of the ratio of senescent leaves / total leaves and the anthocyanin index in concordance with an early decrease of chlorophyll and the Nitrogen balance index. (Figs. 1 and 2).

Leaf senescence is characterized by an active genetic reprogramming process involving many genes, metabolites, and hormones connecting multiple signalling pathways. In this work, utilizing the RNA-seq approach we found almost twice as many genes differentially expressed in the early senescence lines at post-anthesis vs. anthesis sampling times. Moreover, when we compared the list of genes shared between both lines, almost all of them displayed higher (or lower) fold-changes in R453. Additionally, genes related to lipid and protein degradation, and genes related to nutrient recycling were also upregulated indicating an early activation of the senescence program in this line (Fig. 3 and Additional file 3: Table S1).

One of the first symptoms of senescence is a decrease in photosynthesis rate. After anthesis, a decrease in the expression of photosynthesis-related genes was observed in both lines, with lower expression in the early senescence line. Likewise, measurement of photosynthesis activity at 12 days after anthesis showed a higher rate in the delayed senescence line (Fig. 1e).

In different crops, coordinated nutrient recycling is critical to warrant seed growth and affect their quality and quantity. Metabolic profiling revealed a decrease of sugar and TCA cycle metabolites during leaf senescence in sunflower and this decrease was also stronger in the early senescence line (Additional file 1: Figure S1). Similar results were previously reported by our group using a sunflower hybrid VDH487 (Advanta seeds) [19]. Source-sink relationships are mainly governed by sugars [35, 36], and particularly sunflower has high requirements of sugars as a substrate for oil synthesis. Genes associated with sugars and starch degradation, as well as some genes related to cell wall and lipids degradation, were upregulated early at post-anthesis in the early-senescing line indicating an increase of catabolism process (Fig. 4).

It is widely accepted that glutamine and asparagine, are the main amino acids involved in nutrient recycling [37–39]. These amino acids mediates nitrogen and carbon transport between the different organs and are the most abundant amino acids in the xylem and phloem [37, 39, 40]. An accumulation of asparagine levels was found at post anthesis in the early-senescing line (Additional file 1: Figure S1a), demonstrating an active nutrient relocating process in this line. The line B481–6 did not show any differences at the evaluated time points (Additional file 1: Figure S1b). In contrast, low levels of glutamine were detected during leaf senescence in both lines. In sunflower, Agüera et al. [41] showed an increase of asparagine and, in a lower degree, glutamine in senescence leaves under Nitrogen supply. Sink-source relationship and nutrient recycling are an important task for crop improvement. The leaf position and the temporal sampling could affect the metabolite concentration and a deep study using a time-resolved metabolomics analysis may be necessary to understand nutrient recycling in sunflower. Nevertheless, despite steady-state metabolite analyses do not fully represent metabolite translocation fluxes in leaves, this results could be expected to result from both, flux changes and changes of metabolism caused by the sink-source transition [38]. An increase of the amino-acid content was observed during leaf senescence and more pronounced in the early senescing line. This relates to the observed increase of these compounds during drought stress response reported previously by Manivannan et al. [42] and recently by Fernandez et al.*,* [43] and suggests this amino-acid content induction could be a common mechanism shared between developmental and drought-induced senescence.

To understand how these processes are controlled at the gene regulatory level we also studied gene expression modification in the corresponding tissues. Particularly, the ethylene signaling pathway and the TFs associated to this pathway have been widely reported as key elements at triggering and/or controlling the different pathways of senescence process [30, 44, 45]. Thereby, the NAC TF family holds potential candidate genes for senescence control. In Arabidopsis, the NAC TFs ORE1, regulated by ethylene, has been extensively studied as senescence inducer in different plant species [46–48]. In sunflower, we previously reported this gene as a biomarker of leaf senescence available for crop improvement. More recently, Rauf et al. [49] demonstrated that ORE1 is also positively regulated by ATAF1 and that both TFs inhibit the expression of Golden2-like (GLKs) genes, which are necessary for chloroplast development and maintenance. In this study, we found the sunflower orthologous genes to *ORE1* and *ATAF1* with higher expression levels in the early senescence line, even at anthesis time (Fig. 5). Moreover, the GLK gene showed downregulation in this line, indicating a premature activation of the senescence pathway at, or maybe even before, anthesis.

More than 30 NAC genes showed enhanced expression during natural leaf senescence in Arabidopsis [21, 31]. Here, we reported 23 NAC TFs differentially expressed between both lines at anthesis and post-anthesis, most of them upregulated in the early senescence line (Fig. 5). Regarding the TFs differentially expressed during leaf senescence, AP2/ERF, WRKY and MYB families have been reported to have a central role in senescence pathway [32, 33, 45, 50–55]. Among these families, MYB TFs showed the highest differences between lines and most of them were upregulated in early senescence line. Despite displaying differential expression during leaf senescence, only a few members of this family have been functionally reported to trigger senescence [33, 52, 56]. Considering the WRKY TFs family, most of them were downregulated in early senescence line, with it being likely that some of them could negatively regulate the senescence program. The sunflower gene orthologous to *WRKY70* (HanXRQChr09g0252311) was found to be upregulated in delayed senescence line (downregulated in R453) (Fig. 5). This TF, together with WRKY54, has been reported as an negative regulators of the senescence process and, in association with WRKY53, may be closely involved in a regulatory network that integrates internal and environmental factors to modulate the onset and the progression of leaf senescence [32, 57].

In agricultural crops, different methods of co-expression networks analysis have been used to infer gene function providing a powerful tool to understand biological processes [58]. With the aim to identify hub TFs as principal regulator of the senescence gene regulatory network in sunflower, we performed a Weighted Gene Co-expression Network Analysis. We identified 25 hub TFs upregulated in early senescence line at anthesis time (Fig. 6a). Two NAC TFs were detected as hubs, *HanXRQChr02g0048351* and *HanXRQChr05g0156601,* with high sequence similarity to *ANAC082* and *ANAC02/ATAF1.* Recently, Kim et al. [59] reported a NAC TFs network that negatively regulates leaf senescence in Arabidopsis. In this study, ANAC017, ANAC082, and ANAC090, referred as the “NAC troika”, were reported as a negative regulator of leaf senescence showing an increase of gene expression during early leaf senescence. The authors suggest that both, positive and negative senescence regulator genes are upregulated during leaf senescence, which might regulate the initiation and progression of the process at the pre-senescent stage. *ATAF1*, an abscisic acid activated TF*,* was previously reported to be associated with an upstream regulation of the signalling pathway involving ORE1, activating its expression and inhibiting the expression of *Golden2-like (GLKs)* genes by directly binding to their promoters, which are necessary for chloroplast development and maintenance [49, 60].

Interestingly, seven WD40-like genes were found as hubs in the upregulated network (Fig. 6a and Additional file 5: Table S3). The WD40 domains play central roles in a range of biological processes by acting as hubs in cellular networks [61]. The WD40 domain proteins function as an adaptor in many different protein complexes or protein-DNA complexes in very diverse cellular processes [62]. However, only a few of them have been studied. In apple, for instance, *MdATG18a* a WD40-repeat AuTophaGy-related gene, plays a role in responses to leaf senescence and abiotic stresses, indicating autophagy mechanism regulation [63]. Our results suggest that WD40-like family might play a central role in regulating different aspects of the senescence pathway in sunflower.

The *HanXRQChr09g0256311* gene, which had a high number of connections in the co-expression network, has high sequence similarity to MYC4 (a bHLH TF family). MYC4 was identified as a target of JAZ repressors operating with MYC2 in regulating the JA-dependent transcriptional response (Fernandez-Calvo et al., 2011; Niu et al., 2011). More recently, Yu et al. [64] reported that JAZ7 negatively regulates dark-induced leaf senescence by the suppression of MYC2/MYC3/MYC4.

Regarding the downregulated TFs, in this study, we found 36 genes reported as hub genes downregulated in early senescence line (Fig. 6b and Additional file 5: Table S3). Among them, 3 NAC TFs were detected, *HanXRQChr01g0024891*, *HanXRQChr01g0024001,* and *HanXRQChr17g0543751,* with high sequence similarity to *ANAC087, ANAC047* and *ANAC036* respectively. *ANAC087* and *ANAC047* were recently reported to be regulated by the “NAC troika” ANAC090, that negatively regulates leaf senescence, via the direct bound to their promoters [59]. In our study, the sunflower gene orthologous to *ANAC090* (*HanXRQChr13g0397171*) was found to be downregulated at post-anthesis in the early senescence line (Fig. 5). ANAC087 (together with ANAC046) was also reported regulating distinct aspects of programmed cell death in the Arabidopsis columella and lateral root cap during and after its shedding from the root tip [65]. The *ANAC047* gene is activated in an EIN3-independent manner [30], but the role of ANAC047 in leaf senescence has yet to be determined [45].

Zinc finger proteins were also found to be acting hubs in the downregulated network (Additional file 5: Table S3). Zhang et al. [66] identified 1012 genes in wheat that were induced during senescence, reporting an important role of WRKY and zinc finger transcription factors in the early stage of leaf senescence.

It is worth noting that many different TFs were reported as hubs in the networks, providing evidence for suggesting a very complex regulation process. The integration of multi-omics data is necessary to address unsolved questions regarding complex traits like leaf senescence. Especially in crops such as sunflower, where most of the molecular tools for functional analysis of individual genes, such as transgenic or site-directed mutagenesis are not available or difficult to develop, this approach is key to determine the molecular principles that coordinate concurrent and ordered shifts in biological events during leaf senescence [11].

## Conclusions

In summary, in this study we compared the physiological, metabolic and transcriptional changes occurring during leaf senescence using two contrasting inbred lines specifically for this trait. Both lines presented similar phenology and architecture but displayed differences in the progress of senescence once anthesis had started. These differences were also confirmed in very different environments. Metabolic pathway associated with sugars and nutrient recycling were differentially regulated in both lines. Moreover, gene expression profiles showed a higher number of genes, as well as, higher expression levels in the early senescence line, indicating an early activation of senescence program in this line. Additionally, WGCNA analysis reported a large number of TFs as hubs in co-expression networks; some of them were previously reported in different species as senescence-associated genes and but many of them remain to be further characterized.

Understanding the onset and the progress of the senescence process in crops will allow the development of different management strategies to mitigate the senescence impact and the biologically and economically important grain-filling process. The catalog of candidate genes identified in this study remains to be further characterized at a functional level in order to develop molecular tools for biotechnological applications in breeding crop yield.

## Methods

### Plant material and experimental conditions

The experiment was conducted under field conditions at the Biotechnology Institute INTA Castelar during the 2014/15 growing season. Previous selected contrasting inbred lines, R453 (early senescence) and B481–6 (delayed senescence) [20], belonging to the INTA Sunflower Breeding Program, INTA Manfredi Sunflower Germplasm Collection, were used. Plants were sown at 7.2 plants/m^2^. Leaves were sampled periodically, and frozen immediately in liquid nitrogen and stored at − 80 °C until processing. For all the analysis, three biological replicates were used (plots), each one consisted of three randomly selected plants from each plot. Diseases, weeds as well as insects and bird were adequately controlled. Soil fertility assured maximum yields under non-limiting water conditions and soil water was maintained by irrigation. Transcriptomic and metabolic profiles were performed using the 15th leaf (numbered from the bottom to the top of the plant) at two developmental stages, anthesis and post-anthesis time (12 days after anthesis). Time was expressed on a thermal time basis by daily integration of air temperature with a threshold temperature of 6 °C. Also, plant emergence was considered as thermal time origin (°CDAE: *°C Days After Emergence*) as previously proposed for sunflower [67]. With the aim to test if these contrasting inbred lines are able to reproduce the differences in leaf senescence in a different environment, we performed a phenotyping assay in the Heliaphene Platform at LIPM - INRA Toulouse, France, during 2017 growing season.

### Physiological measurements

Different physiological and phenotypic measurements were assessed during the experiment:

Chlorophyll content was measured by chemical extraction with ethanol. 50 mg of ground tissue was incubated 24 h in the dark with 1.5 ml of ethanol 95% (v / v) until all pigments were completely dissolved. The absorbance of each sample was measured using a 96-well spectrophotometer and chlorophyll content was calculated as [68]:

$$ \mathrm{Chl}\ \left(\upmu \mathrm{g}/\mathrm{ml}\right)=5.24\ast \mathrm{A}664+22.24\ast \mathrm{A}649. $$

$$ \mathrm{Chl}\ \left(\upmu \mathrm{g}/\mathrm{mg}\ \mathrm{FW}\right)=\mathrm{Chl}\ \left(\upmu \mathrm{g}/\mathrm{ml}\right)\ast 1.5\ \mathrm{ml}\ \left(\mathrm{ethanol}\right)/\mathrm{FW}\Big). $$

Nondestructive measurements were periodically performed in three randomly selected plants from each plot (*n* = 9). Evolution of leaf area was calculated as Casadebaig et al. [69]:

$$ {\displaystyle \begin{array}{l} If\kern4.5em \left(L\ x\ W\right)<\frac{c}{a\hbox{-} b}\kern4em \\ {} else\end{array}}{\displaystyle \begin{array}{l}S=a\ \mathit{\mathsf{X}}\left(L\ \mathit{\mathsf{X}}\ W\right)\\ {}S=b\ \mathit{\mathsf{X}}\left(L\ \mathit{\mathsf{X}}\ W\right)+c\end{array}} $$

where L = leaf length and W = maximum width, a = 0.684, b = 0.736, c = − 8.860.

Quantum yields (QY) of photosystem II in the 15th leaf was periodically measured using a fluorometer FluorPen FP 100 (Photon Systems Instruments) and radiation interception of the canopy through a radiation bar ACCUPAR LP-80 (METER Environment). Likewise, 12 days after anthesis, measurements of photosynthetic activity were carried out using a LI-COR LI-6400XT Portable Photosynthesis System (Instrumentalia S.A.).

Using the Heliaphen platform [28], we performed measurements of total leaf number, ratio of senescence – total leaf number and time to anthesis. Moreover, chlorophyll and anthocyanin content as well as Nitrogen balance index (NBI) was quantified on leaf 15 periodically using a Dualex Scientific+ (FORCE-A, France).

### Transcriptomic analysis

#### RNA isolation, quantification, and quality controls

Leaves were frozen immediately in liquid nitrogen upon collection and stored at − 80 °C until processing. High quality total RNA was isolated from 100 mg of frozen tissue using TriPure, according to the manufacturer’s instructions (Roche, Buenos Aires, Argentina). Genomic DNA was eliminated after treatment with DNase I for 20 min at room temperature using DNase I (Invitrogen, Argentina). The RNA concentration was measured using a Qubit fluorometer (Invitrogen, Argentina). The purity and integrity of total RNA were determined by Fragment Analyzer (Advanced Analytical Technologies, USA).

#### RNA-seq assay

RNA libraries were performed using the kit TruSeq Stranded mRNA Technology (Illumina). All the libraries were evaluated according to standard quality controls of the Consorcio Argentino de Tecnologías Genómicas (CATG). Each library was pair-end sequenced (2 × 100 pb) with a depth of 20 million reads [70] using a HiSeq-1500 (Illumina) (NCBI - SRA accession: PRJNA525834).

Quality control of reads was evaluated using FastQC and trimmed or filtered using trim_galore. Reads were aligned to the reference genome [27] using Bowtie2 software with parameters: global alignment and to keep all multi-mapping reads. After obtaining all read mapping files (BAM files), we performed re-estimation of all counts to all transcripts using the eXpress software which uses an EM method for estimation of multi-mapping reads. To perform statistical analysis of counts, we used the effective counts, which are the estimated number of fragments generated from this target in the sequencing experiment, adjusted for fragment and length biases. Statistical and differential gene expression analysis was performed using DESeq package [71] in R.

#### qPCR assay for RNAseq validation

The expression of selected genes was evaluated in relation to Elongation Factor -1α (EF-1α), which has been previously selected as a reference gene for expression leaf senescence experiments [72] (Additional file 2: Figure S2). Specific primer pairs were designed using Primer3 software [73], under default parameters.

For each sample, 500 ng of RNA was treated with DNase and reverse-transcribed (Superscript III first strand synthesis system, Invitrogen, Buenos Aires, Argentina) using random hexamer primers according to the manufacturer’s instructions as previously reported in sunflower [18]. qPCR was carried out using FastStart Universal SYBR Green Master Mix (Roche Applied Science) in an ABI StepOne thermocycler (Applied Biosystems). Amplification efficiencies and Ct values were determined using the LinRegPCR [74]. Gene relative expression was calculated using fgStatistic software [75] based on previously published algorithms [76].

### Metabolic analysis

#### GC-TOF-MS analysis

Metabolite extraction was performed via the extraction of lipophilic and polar compounds according to Roessner-Tunali et al. [77] with adaptations to sunflower tissue [15]. Samples were derivatised and injected (1 μl) into the GC-TOF-MS system (LECO Corporation, St. Joseph, Michigan, USA). Chromatography was performed on a 30 m SPB-50 column with 0.25 mm inner diameter and 0.25 lm film thickness (Supelco, Bellefonte, CA, USA). The temperatures of injection, interface, and ion source were set to 230 °C, 250 °C, and 200 °C, respectively. The carrier gas was He at a constant flow ratio of 1 ml.m^− 1^. The chromatograms and spectra were evaluated using the ChromaTOF (LECO Corporation, St. Joseph, Míchigan, USA) and TagFinder [78]. Ion spectra were compared to the Golm Metabolome Database (http://gmd.mpimp-golm.mpg.de/). Metabolite levels were normalized to fresh weight and the internal control ribitol. Changes in metabolite levels were calculated as the fold-change relative to control conditions.

### Transcriptomic and metabolic integration

#### MapMan analysis

MapMan software [29] was used to integrate transcriptomic and metabolic profiles. The sunflower transcriptome [27] was annotated via the Mercator annotation pipeline [79] and used as a library in Mapman.

The expression ratio cut-off was log2 fold change higher or lower than 2 (− 2) and with a *p*-value lower than 0.05. The resulting data table was used for MapMan analysis.

#### Weighted gene co-expression network analysis (WGCNA)

WGCNA was performed using the WGCNA R package (v1.63) as described by Langfelder and Horvath [80]. The expression values (rld normalized in DESeq) for 38,775 filtered and non-redundant genes were used to construct the network using the following settings: power = 5, minModuleSize = 50, mergeCutHeight = 0.2, maxBlockSize = 40,000, deepSplit = 2, reassignThreshold = 1e− 6 and minKMEtoStay = 0.3, networkType = “signed”, TOMType = “signed”. The network modules were correlated with metabolite levels and differentially expressed transcription factors. The network was exported for all 21 modules using the function exportNetworkToCytoscape (edge weight threshold of > = 0.4) giving a network of 9538 nodes and 9,247,259 edges. A total of 449 transcription factors differentially expressed between both lines at anthesis time were selected from this network, being 322 out of 449 genes present in the network, so this produced a network of 322 nodes and 16,785 edges. This network was further filtered using the list of up and down-regulated genes with a Log2 fold-change > 2 or < − 2 before visualization using Cytoscape [81].

## Supplementary information

**Additional file 1: Figure S1.** Metabolites profile view using Mapman. (a) R453 line and (b) B481–6 line. Color intensity corresponds to the expression ratio at logarithmic scale (red: up-regulated, blue: down-regulated) of Post-anthesis vs. Anthesis for each line.

**Additional file 2: Figure S2.** qPCR assay for RNAseq validation. Expression levels on a logarithmic scale (log2) of selected genes (named according to the identification number in the Heliagene XRQ Genome Portal) Post-anthesis vs. Anthesis for each line and by using the elongation factor 1α (HaEF1α) as the reference gene. Green bars correspond to B481–6 line and orange bars correspond to R453 line.

**Additional file 3: Table S1.** Differentially expressed genes between post-anthesis vs. anthesis in the early and delayed senescence lines respectively.

**Additional file 4: Table S2.** Metabolites profile between post-anthesis vs. anthesis in the early and delayed senescence lines respectively.

**Additional file 5: Table S3.** Expression level and degree of TFs up- and down-regulated selected by WGCNA analysis.

## Data Availability

All data generated or analysed during this study are included in this published article and its additional files. RNAseq data are available at NCBI - SRA accession: PRJNA525834.

## Abbreviations

CdAE: C Days After Emergence
GC-TOF-MS: Gas chromatography - time-of-flight - mass spectrometry
GLA: Green leaf area
GLKs: Golden2-like genes
NBI: Nitrogen balance index
NGS: Next-Generation Sequencing
TCA cycle: Tricarboxylic acid cycle
TFs: Transcription Factors
WGCNA: Weighted Gene Co-expression network analysis
