# Supplementary figures and images for: Exploring gene networks in two sunflower lines with contrasting leaf senescence phenotype using a system biology approach
Source: BMC Plant Biol. 2019 Oct 24;19:446. doi: 10.1186/s12870-019-2021-6 (PMC6813990; doi:10.1186/s12870-019-2021-6)

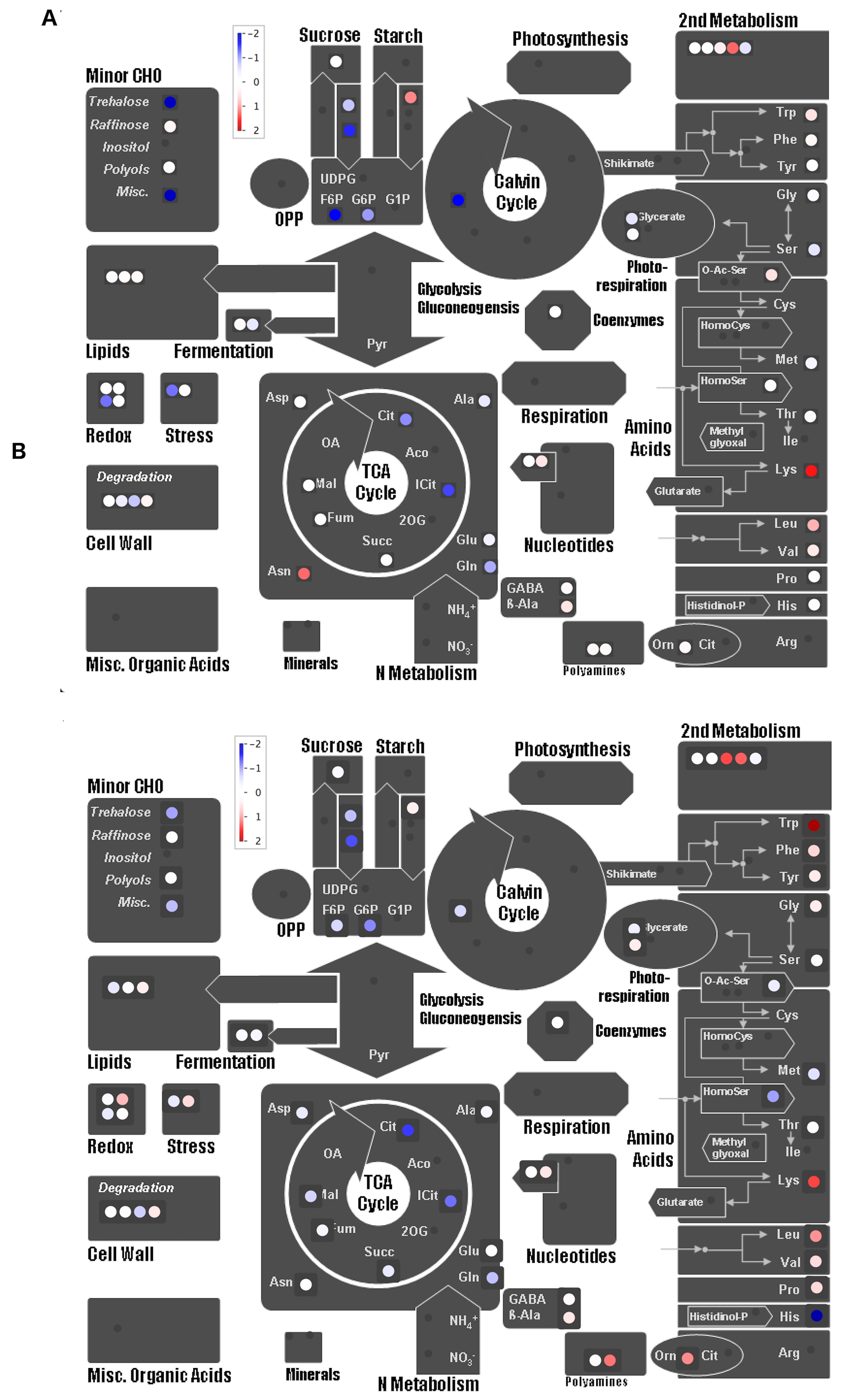

Supplement: Supplementary file 1 — Additional file 1: Figure S1. Metabolites profile view using Mapman. (a) R453 line and (b) B481–6 line. Color intensity corresponds to the expression ratio at logarithmic scale (red: up-regulated, blue: down-regulated) of Post-anthesis vs. Anthesis for each line. [file 12870_2019_2021_MOESM1_ESM.png]

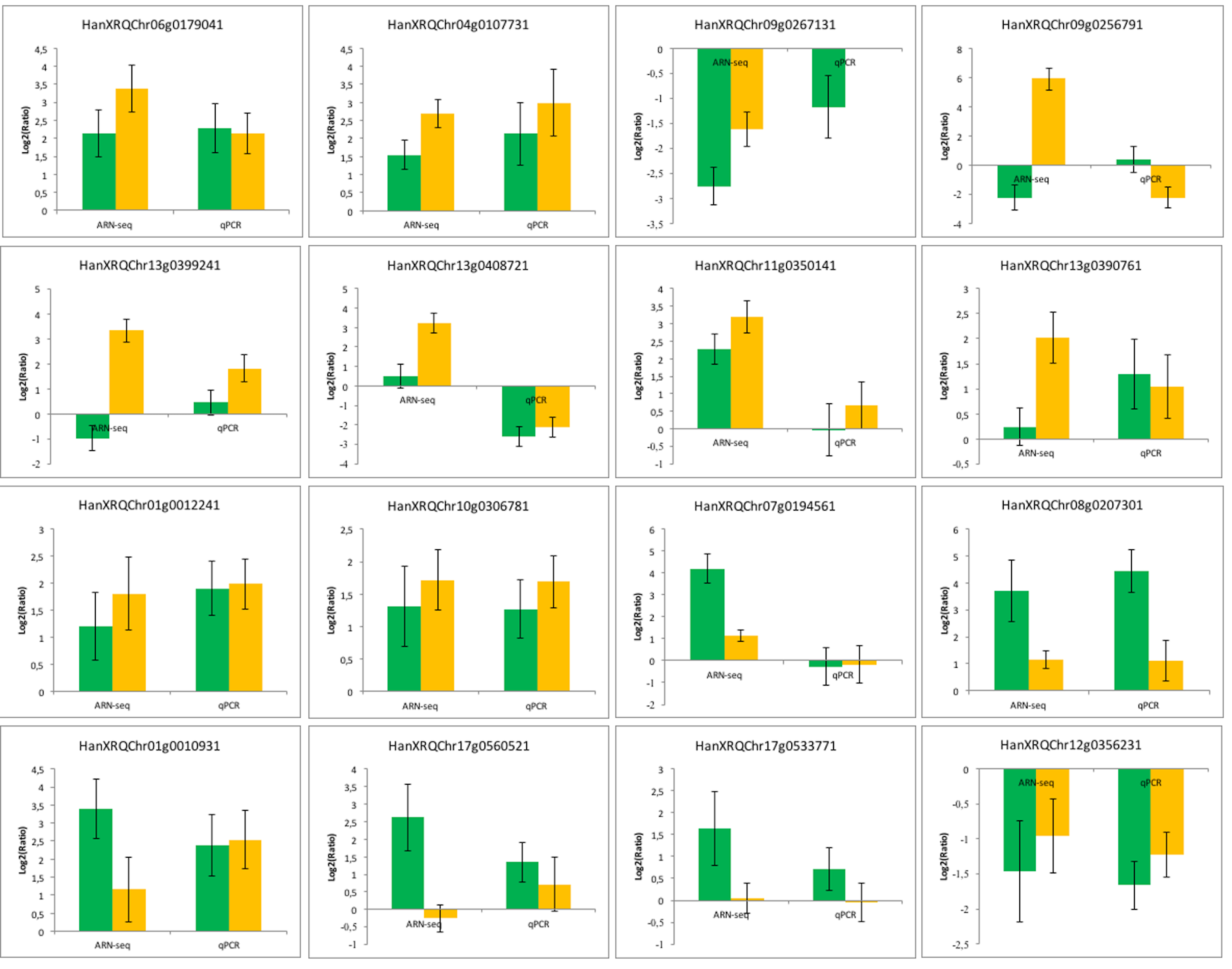

Supplement: Supplementary file 2 — Additional file 2: Figure S2. qPCR assay for RNAseq validation. Expression levels on a logarithmic scale (log2) of selected genes (named according to the identification number in the Heliagene XRQ Genome Portal) Post-anthesis vs. Anthesis for each line and by using the elongation factor 1α (HaEF1α) as the reference gene. Green bars correspond to B481–6 line and orange bars correspond to R453 line. [file 12870_2019_2021_MOESM2_ESM.png]
